# Everolimus and pazopanib (E/P) benefit genomically selected patients with metastatic urothelial carcinoma

**DOI:** 10.1038/s41416-018-0261-0

**Published:** 2018-09-17

**Authors:** Joaquim Bellmunt, Aly-Khan A. Lalani, Sussana Jacobus, Stephanie A. Wankowicz, Laura Polacek, David Y. Takeda, Lauren C. Harshman, Nikhil Wagle, Irene Moreno, Kevin Lundgren, Dominick Bossé, Eliezer M. Van Allen, Toni K. Choueiri, Jonathan E. Rosenberg

**Affiliations:** 1 Department of Medical Oncology, Dana-Farber Cancer Institute, Boston, MA USA; 2 Htal Del Mar Research Institute-IMIM, Barcelona, Spain; 3 Juravinski Cancer Centre, McMaster University, Hamilton, Canada; 4 Department of Biostatistics and Computational Biology, Dana-Farber Cancer Institute, Boston, MA USA; 5 The Eli and Edythe L. Broad Institute of Harvard and MIT, Cambridge, MA USA; 6 Fundación Jiménez Díaz University Hospital, Madrid, Spain; 7 Department of Medicine, Memorial Sloan-Kettering Cancer Center, New York, NY USA

## Abstract

**Background:**

Metastatic urothelial carcinoma (mUC) is a genomically diverse disease with known alterations in the mTOR pathway and tyrosine kinases including FGFR. We investigated the efficacy and safety of combination treatment with everolimus and pazopanib (E/P) in genomically profiled patients with mUC.

**Methods:**

mUC patients enrolled on a Phase I dose escalation study and an expansion cohort treated with E/P were included. The primary end point was objective response rate (ORR); secondary end points were safety, duration of response (DOR), progression-free survival (PFS) and overall survival (OS). Patients were assessed for mutations and copy number alterations in 300 relevant cancer-associated genes using next-generation sequencing and findings were correlated with outcomes. Time-to-event data were estimated with Kaplan–Meier methods.

**Results:**

Of the 23 patients enrolled overall, 19 had mUC. ORR was 21% (one complete response (CR), three partial responses (PR), eight with stable disease (SD). DOR, PFS and OS were 6.5, 3.6, and 9.1 months, respectively. Four patients with clinical benefit (one CR, two PR, one SD) had mutations in TSC1/TSC2 or mTOR and a 5th patient with PR had a FGFR3–TACC3 fusion.

**Conclusions:**

Combination therapy with E/P is safe in mUC and select patients with alterations in mTOR or FGFR pathways derive significant clinical benefit.

## Background

While platinum-based chemotherapy has served as the backbone of treatment for patients with metastatic urothelial carcinoma (mUC), these patients often progress after first-line therapy and outcomes thereafter have been poor (https://seer.cancer.gov/statfacts/html/urinb.html). Due to the rich genomic diversity of mUC, molecular profiling provides an opportunity to better characterise this disease and ultimately assist in the development of rational therapeutic options (https://www.mycancergenome.org/content/disease/bladder cancer/). Genomic profiling has revealed potential pathways for targeted anticancer treatment in mUC.^1–4^ While targeted therapies have led to improved outcomes in multiple tumour types, the role of these agents has not yet been standardised in mUC.^5^

Two potential avenues for targeted therapy in mUC are the vascular endothelial growth factor (VEGF) and mammalian target of rapamycin (mTOR) pathways. Angiogenesis via VEGF is known to play a role in bladder cancer biology and disease progression^6^ and activation of mTOR has been shown to increase tumour cell proliferation and promote angiogenesis.^7^ This understanding has led to investigations into the potential use of VEGF tyrosine kinase inhibitors (VEGF-TKIs) and mTOR inhibitors as combination therapy. Preclinical models have shown this combination to augment both antiangiogenic and antitumour effects in a synergistic fashion.^8,9^ Early clinical studies have attempted to elucidate the efficacy and safety of this potential combination in a variety of cancers;^10–13^ however, the paucity of data in mUC presents an unmet need.

The combination of the mTOR inhibitor everolimus (E) and VEGF-TKI pazopanib (P) has been evaluated in a phase I clinical trial of patients with advanced solid tumours. We previously reported on the initial results of this trial including a patient described as an 'extreme responder'.^14^ Subsequently, an expansion cohort of patients with mUC was added (NCT01184326). In this analysis, we report the safety and efficacy of combination treatment with E/P, as well as the associations between clinical outcomes and molecular alterations, in genomically profiled patients with mUC enrolled on this phase I study.

## Methods

### Patients

Eligible patients for the dose escalation cohort were 18 years or older, had Eastern Cooperative Oncology Group (ECOG) performance status 0–1, normal organ and marrow function, and metastatic or unresectable solid tumours for which standard curative or palliative measures do not exist, are not tolerable, or are no longer effective. Patients who received prior everolimus or pazopanib therapy or those with unstable medical conditions were also excluded, as previously described.^14^ For the expansion cohort, key eligibility criteria include: metastatic or locally advanced unresectable urothelial carcinoma with pathological confirmation; measurable disease as defined by Response Evaluation Criteria in Solid Tumours (RECIST) guidelines version 1.1;^15^ and, previous treatment with at least one, but not more than 3, lines of systemic chemotherapy including either a platinum agent, a taxane, or gemcitabine.

### Study design

We conducted a phase I study with an expansion cohort in patients with advanced urothelial cancer, as reported previously.^14^ A standard '3+3' dose escalation design was used to determine the maximum tolerated dose (MTD) of the combination of everolimus and pazopanib in patients with solid tumours who have no standard treatment options available. Starting doses were set at everolimus 5 mg and pazopanib 400 mg taken orally, daily with treatment self-administered by each patient. Each cycle was 4-weeks (28 days) duration. Two dose levels (DLs) for everolimus (E) and three DLs for pazopanib (P) and its combinations (E/P) were evaluated as: E 5 mg daily + P 400 mg daily (DL-1); E 5 mg + P 600 mg (DL0); E 10 mg + P 600 mg (DL-1); E 10 mg + P 800 mg (DL2); and, E 5 mg daily + P 800 mg daily (DL2A, only used if the MTD was exceeded on DL2). Initial phase I testing identified everolimus 5 mg and pazopanib 400 mg as the MTD; these doses were used for combination therapy in the dose expansion cohort of patients with advanced urothelial cancer. Treatment continued until disease progression, unacceptable toxicity, clinical deterioration or participant withdrawal from the study.

The study was conducted with Dana-Farber/Harvard Cancer Center institutional review board approval in accordance with the Declaration of Helsinki and Good Clinical Practice and was registered with the National Institutes of Health (NCT01184326). Informed consent was obtained from patients.

### Outcomes

The primary objective for the dose expansion cohort was to determine the objective response rate (ORR) in patients with locally advanced or metastatic urothelial cancer (mUC) treated with the combination of everolimus and pazopanib (E/P). Tumour assessments were performed at baseline and every 8 weeks thereafter with responses or progression evaluated by RECIST criteria, v1.1 using conventional computed tomography (CT) or magnetic resonance imaging (MRI) of the chest, abdomen and pelvis. Each participant was assigned one of the following categories: complete response (CR); partial response (PR); stable disease (SD); progressive disease (PD); and, unknown if not evaluable or insufficient data. Only eligible patients who start protocol therapy were included in the response analysis.

Secondary objectives were to determine the safety, duration of response (DOR), progression-free survival (PFS) and overall survival (OS) of the combination treatment. Safety assessments included adverse events (classified and graded according to the National Cancer Institute Common Terminology Criteria for Adverse Events version 4.0, CTCAE v4.0), physical examinations, ECOG scores, laboratory tests (urinalysis, complete blood count and comprehensive metabolic panel performed every 2 weeks during the first two cycles and then once every 4 weeks), and electrocardiogram (ECG, performed for assessment of the corrected QT interval with the first two cycles). Dose-limiting toxicity (DLT) was also documented with evaluation methodology as previously described.^14^

Toxicity incidence was tabulated by maximum grade for a given toxicity type including only events with an attribution of possibly, probably or definitely treatment related. Unevaluable patients were counted in the denominator of the ORR. DOR was defined as the time in months from the first observation of objective response (PR or better) to the first-documented progression or death due to any cause, whichever occurred first. PFS was calculated as the time in months between registration and documented PD as determined by RECIST 1.1, or death due to any cause, whichever occurred first. Patients alive without disease progression were censored at the date of last disease evaluation. OS was defined as the time in months from registration to death due to any cause or censored at the date last known alive.

### Genomic analysis

Deep-targeted next-generation sequencing was performed using Dana-Farber Cancer Institute PROFILE test, a hybrid-capture and massively parallel sequencing assay surveying exonic DNA of 400 cancer genes as reported previously.^16^ Patient cystectomy tissue, or related bladder surgical sampling (i.e., TURBT), were interrogated. Findings were correlated with clinical benefit.

Computational algorithms that predict neutral, detrimental or activating variants were used. Relevant associations between mutation status and treatment outcome were assessed on an exploratory basis.

### Statistical plan

Once the recommended dose for E/P was established, planned enrolment was for 29 patients in the expansion cohort. There was a two-stage design employed to evaluate the expansion cohort. The planned first stage was to accrue 20 patients (yielding 18 eligible patients) and if three or more patients achieved an objective response, then an additional nine patients would be accrued (yielding eight eligible patients). The combination treatment of E/P would be considered effective if five or more responses were observed in the 26 eligible patients who started protocol therapy. This study was designed to have 90% power to discern between an ORR of 10% versus 30% with a one-sided type I error of 10%. The cutoff date for final analysis was March 31, 2017.

Patient and clinical characteristics were summarised as numbers and percentage for categorical variables and median with range for continuous variables. Time-to-event data were estimated with the Kaplan–Meier method.

## Results

### Patients

Overall, 23 patients were consented for trial enrolment and received treatment with E/P: nine patients in the dose-finding cohort (from January 2011 to September 2011) and 14 patients in the expansion cohort (from march 2015 to June 2016). The study was terminated early due to slow accrual. The primary sites were bladder cancer (*n* = 19), non-small-cell-lung cancer (*n* = 3) and adrenocortical carcinoma (*n* = 1). Median follow-up time was 22 months. Evaluation of patients is reported through March 31, 2017. We describe the results for the 19 patients with mUC (5 patients from the dose-finding cohort and all 14 in expansion cohort). Baseline characteristics are shown in Table 1. In this group, the median age was 69 years, 17 (89.5%) were male and 18 (94.7%) were identified as white race. Thirty-nine percent of patients had visceral metastatic disease. All patients had experienced disease progression on standard therapies and 69% received one prior line of therapy, 31% more than one. Median duration of treatment was 3.7 months (range, 0.3–13.8 months) and median number of cycles completed was 4 (range, 0–13).

**Table 1:** Baseline characteristics for metastatic urothelial carcinoma patients

Characteristic, *n* (%)	Patients with metastatic urothelial carcinoma (*n* = 19)
Age, median years (range)	69 (50–88)
Sex	
Male	17 (89.5%)
Female	2 (10.5%)
Race	
White	18 (94.7%)
Asian	1 (5.3%)
Dose group	
Dose level 0 (phase I)	3 (15.8%)
Dose level −1 (phase I+expansion)	16 (84.2%)
Number of prior treatments	
1 prior therapy	9 (69.2%)
>1 up to 3 prior therapies	4 (30.8%)
Unknown	6

### Efficacy

For the 19 patients with mUC, 16 had evaluable disease and ORR was 21%. One patient demonstrated CR, three patients had PR and eight had SD. Four patients were found to have PD at the time of evaluation. For the four responders (one CR, three PR), mean DOR was 6.7 months and, specifically, 12.0, 7.4, 5.6 and 1.7 months, respectively. Overall, median PFS was 3.6 months (95% confidence interval (CI): 1.8–5.6 months) and 12-month PFS was 5.6% (95% CI: 0.4–22.7%). Median OS was 9.1 months (95% CI: 6.2–13.1 months) and 12-month OS was 34.2% (95% CI: 14.2–55.5%).

### Safety

For the Phase I dose-finding cohort, three patients were enrolled on the initial dose-level DL0. One patient was ineligible for DLT assessment due to an unscheduled break in therapy during cycle 1, unrelated to treatment. Among eligible patients, one patient experienced DLT in the first cycle of treatment related to rash (grade 3) and serum hyperuricemia (grade 4) and another patient experienced DLT related to thrombocytopenia (grade 3). Subsequently, three patients were enrolled to DL-1 level and no DLTs were observed. An additional three patients were treated at this dose-level (DL-1) per protocol to evaluate for MTD and no further DLTs were observed. Therefore, the study continued to the expansion cohort with 14 more patients treated at this dose level.

In the cohort of patients with mUC, 18 (94.7%) patients experienced an adverse event (AE) of any grade. The most common all-grade AEs were hypophosphatemia, diarrhoea, fatigue, thrombocytopenia and hyperglycaemia (Table 2). Electrolyte disturbances were most often asymptomatic and transient. Grade 3 or higher toxicity was reported in 14 (73.7%) patients. One patient experienced grade 5 AE attributed to an intracranial haemorrhage. Reasons for treatment discontinuation are listed in Table 3.

**Table 2:** Selected adverse events in patients with metastatic urothelial carcinoma

All patients with metastatic urothelial carcinoma (*n* = 19)				
	Grades 1–2	Grade 3	Grade 4	Grade 5
Non-haematological				
Diarrhoea	8 (42%)	1 (5%)	0	0
Nausea	3 (16%)	0	0	0
Anorexia	4 (21%)	0	0	0
Fatigue	5 (26%)	2 (11%)	0	0
Oral mucositis	3 (16%)	1 (5%)	0	0
Pancreatitis	0	1 (5%)	0	0
Dysgeusia	3 (16%)	0	0	0
Hypertension	2 (11%)	0	0	0
Rash (acneiform)	4 (21%)	0	0	0
Rash (maculopapular)	0	1 (5%)	0	0
Pneumonitis	3 (16%)	0	0	0
Intracranial haemorrhage	0	0	0	1 (5%)
Pneumothorax	0	1 (5%)	0	0
Pruritis	0	1 (5%)	0	0
Weight loss	4 (21%)	0	0	0
Elevated AST	2 (11%)	2 (11%)	0	0
Elevated ALT	4 (21%)	0	0	0
Hypercholesterolaemia	2 (11%)	0	0	0
Viral hepatitis	0	1 (5%)	0	0
Hyperglycaemia	5 (26%)	0	0	0
Hypertriglyceridemia	4 (21%)	0	0	0
Hyperuricemia	0	0	1 (5%)	0
Hypomagnesemia	4 (21%)	0	0	0
Hypophosphatemia	6 (31%)	4 (21%)	0	0
Elevated lipase	1 (5%)	1 (5%)	0	0
Hyponatremia	1 (5%)	1 (5%)	0	0
Haematological				
Anaemia	2 (11%)	0	0	0
Neutropenia	0	1 (5%)	0	0
Leukopenia	2 (11%)	0	0	0
Lymphopenia	0	0	1 (5%)	0
Thrombocytopenia	5 (26%)	2 (11%)	0	0

Selected grades 1–2 (in at least 10% of patients) and grades 3, 4, and 5 adverse events

*AST* aspartate aminotransferase, *ALT* alanine aminotransferase

**Table 3:** Reason for treatment discontinuation for metastatic urothelial carcinoma patients receiving everolimus and pazopanib combination at dose levels 1 and 0 (DL-1 and DL0)

Reason for discontinuation	Treatment DL-1 (*n* = 16) *N* (%)	Treatment DL0 (*n* = 3) *N* (%)	Total (*n* = 19) *N* (%)
Adverse event/toxicity	—	1 (33.3)	1 (5.3)
Progressive disease	14 (87.5)	1 (33.3)	15 (78.9)
Death	1 (6.3)	—	1 (5.3)
Physician decision	—	1 (33.3)	1 (5.3)
Patient withdrawal	1 (6.3)	—	1 (5.3)

### Genomic correlates

Genomic alterations observed in the cohort of patients with mUC are displayed in Table 4. Four patients that derived clinical benefit from combination treatment with E/P (one CR, two PR and one SD) had mutations in mTOR or TSC1/TSC2. Specifically, the patient demonstrating CR had activating mTOR mutations (*E2419K* and *E2014K*); these findings have been reported in detail previously.^14^ Two patients (one PR and one SD) were found to have separate mutations in TSC1 (patient with PR having c.1579C>T (p.Q527*), exon 15 in 27% of 209 reads; patient with SD having c.1237C>T (p.Q413*), exon 12 in 90% of 74 reads). Another patient with PR was shown to have molecular alterations in mTOR plus two DNA variants, EP300 (c.2050_2053+delTCTAG) and KDM6A (c.4187_4191delTACCA), as well as deletion in exon 1 of ARID1A and amplitude gains in MDM2 and CCNE1.

**Table 4:** Genomic alterations on potential targets for everolimus and pazopanib in patients with metastatic urothelial carcinoma

Patient BR	mTOR	TSC1	TSC2	PTEN	AKT1	PIK3CA	FGFR1	FGFR2	FGFR3	FGFR4	VHL	KIT	PDGFRA	PDGFRB
CR (ref 14)	E2014K E2419K													
PR		C.1579C>T (p.Q527)												
PR									FGFR3–TACC3 fusion					
PR	c.3654+3G>A						Single copy del: 8p11.23							
SD		Single copy del: 9q34.13			Single copy del: 14q32.83				c.2315C>T (p.P772L)	Single copy del: 5q35.2				Single copy del: 5q32
SD		c.1237C>T (p.Q413^a^)				Low copy gain: 3q26.32				Single copy del: 5q35.2	Low copy gain: 3p25.3			Single copy del: 5q32
PD		Single copy del: 9q34.13		Single copy del: 10q23.31	Low copy gain: 14q32.33			single copy del: 10q26.13	Single copy del: 4p16.3	c.1054C>G (p.P352A)	Low copy gain: 3p25.3	Low copy gain: 4q12	Low copy gain: 4q12	Single copy deletion: 5q32
PD						Single copy del: 3q26.32	Low copy gain: 8p11.23							
PD		Single copy del: 9q34.13				Single copy del: 3q26.32				Single copy del: 5q35.2				Single copy del: 5q32

*BR* best response, *PR* partial response, *PD* progressive disease, *SD* stable disease, *CR* complete response

^a^Alterations listed reflect PROFILE testing results, except one patient who had whole-exome sequencing (CR, ref 14). Seven additional patients (one PD and six SD) did not have genomic profiling performed

In addition, molecular analysis on a fifth patient with clinical benefit from combination E/P treatment (PR) demonstrated the presence of an FGFR3–TACC3 fusion, without alterations in the mTOR pathway. Patients who only demonstrated progressive disease as best response (*n* = 4) as well as the remaining patients with SD (*n* = 7) were not found to have mTOR or FGFR pathway alterations, and no other relevant driver mutations were noted (Table 4).

## Discussion

In this phase I study with an expansion cohort of 19 patients with mUC, we demonstrate that combination therapy with E/P is safe and potentially efficacious. We previously reported on the dose-finding results of the initial phase I study, whereby clinical safety demonstrated that E/P should be combined at doses 50% lower than their standard doses to 5 mg and 400 mg daily, respectively.^14^ This was in the context of pharmacokinetic drug interactions between the two drugs showing an apparent decrease in everolimus clearance when combined with pazopanib. Therefore, this dose level was established for use in our expansion cohort of 14 additional patients with mUC.

Combination therapy with E/P was generally well tolerated for mUC patients at this dose. Toxicities experienced in our study are consistent with those commonly seen with these two drug classes and no new safety signals were identified (Table 2). In our cohort of previously treated patients with mUC, the ORR was 21% with PFS and OS of 3.6 and 9.1 months, respectively. While this study is an early phase trial using targeted therapies, the response rates seen with E/P are relatively similar to the efficacy of currently approved second-line chemotherapy agents in mUC, of which none is a preferred option over another.^17–22^ Given that exposure to first-line chemotherapy may render patients unsuitable for subsequent-line systemic cytotoxic treatment, the choice of an optimal agent in this space remains an important need. In this context, single agent immune checkpoint blockade with anti-PD-1/PD-L1 antibodies has rapidly emerged as standard of care in the United States (US) for patients following progression on platinum-based chemotherapy, and is becoming an option as first-line therapy for cisplatin-ineligible patients.^23–27^ Overall, similar response rates have been seen with immunotherapy options in the second-line setting of 15–25%, suggesting that the majority of patients will, however, not respond to these treatments.^28^ Further, only one agent (pembrolizumab) has been shown to extend OS in the phase III Keynote-045 trial, with the IMvigor211 trial failing to demonstrate a survival benefit for atezolizumab.^25,29^ Therefore, while immune checkpoint blockade has represented a major breakthrough for some patients with advanced urothelial carcinoma, continued investigations of combination targeted therapies—such as E/P—have the potential to provide further viable therapeutic options for these patients, particularly in the context of relevant genomic profiling.

The rationale for investigating targeted therapies in mUC is derived from the emerging data of molecular analysis that has helped further characterise this disease (https://www.mycancergenome.org/content/disease/bladder-cancer/).^1–4,30^. Of particular interest are mutations in the mTOR pathway and the tyrosine–kinase fibroblast growth factor receptor (FGFR). For example, in a study of comprehensive genomic profiling of recurrent or metastatic UC cases, TSC1 mutations were seen in up to 9.5% of samples and alterations of FGFR3 in up to 21%.^31^ In our study, five subjects that demonstrated clinical response carried mutations in the mTOR pathway, TSC1/TSC2, or were found to have FGFR3–TACC3 fusion (Table 4). Our findings are generally consistent with previous reports of these molecular alterations and should be interpreted in the context of the sample size and the targeted panel-sequencing platform used. Mutations in the tumour suppressor genes TSC1 and TSC2 are well-described activating mutations in the kinase domain of the mTOR pathway. The TSC1/TSC2 complex, through its GTPase-activating protein activity towards the small G-protein Rheb, is a critical negative regulator of mTOR complex 1 (mTORC1).^32^ Inactivating mutations of TSC1/TSC2 result in mTOR pathway activation and these alterations have been shown, collectively, to confer sensitivity to mTOR inhibitors in patients with hamartomatous syndromes, such as tuberous sclerosis complex.^33–35^ These findings may explain the response to E/P seen in our mUC patients harbouring inactivating mutations in TSC1/TSC2 or activating mutations in mTOR.^14^

FGFR3 is a member of a structurally related family of tyrosine kinase receptors that orchestrate a diverse variety of cellular activities, including proliferation, differentiation and survival.^36^ While the *FGFR3* gene is one of the most frequent genetic alterations seen in bladder cancer, aberrant activation is also seen in chromosomal rearrangements of FGFR3 with potential fusion partners, such as TACC3 (transforming acid coiled coil 3).^4^ FGFR3–TACC3 translocations generate constitutively activated and oncogenic FGFR3 kinase protein products. Cellular dependence on these drivers confers sensitivity to selective FGFR inhibition, thereby making it an attractive actionable target in bladder cancer.^37–40^ Pazopanib, a multikinase inhibitor targeting FGFR, VEGFR1-3, platelet-derived growth factor (PDGFR), and c-KIT, has shown promise in mUC particularly in patients with *FGFR* gene amplifications.^41^ Additionally, the combination of pazopanib and everolimus has been shown to be effective in a patient with *FGFR* gene rearrangements.^31^ This may explain why our patient with FGFR3–TACC3 fusion was sensitive to combination E/P therapy and displayed a PR, despite having previously progressed on chemotherapy. Further, the potential synergistic effects of E/P cannot be excluded and, therefore, appropriate interpretation of granular molecular alteration reporting will be important to truly understand precise driver mutations at an individual patient level. Collectively, our correlative findings provide further evidence supporting the clinical benefit of combination mTOR and FGFR-directed therapies in genomically classified patients with mUC, and suggests that further studies in this setting are warranted. Ongoing trials evaluating BGJ398, a pan-FGFR kinase inhibitor, in patients with advanced solid tumours may be informative (NCT01004224 and NCT02160041).

In conclusion, we demonstrate that combination therapy with E/P is well tolerated in patients with mUC and that genomically selected patients with mutations in the mTOR pathway or FGFR appear to derive significant clinical benefit. Investigations are warranted to evaluate combination treatment of mTOR inhibitors and VEGF-TKIs in mUC, particularly given the current landscape of therapies available for those having progressed on platinum-based chemotherapy. Furthermore, our study emphasises the potential for genomically driven clinical trials to help identify novel molecular mechanisms and therapeutic targets.

## **Availability of data and material**

All data and materials are available upon request.
